# A Methodology for Dynamic Assessment of Laboratory Safety by SEM-SD

**DOI:** 10.3390/ijerph18126545

**Published:** 2021-06-17

**Authors:** Laihao Ma, Xiaoxue Ma, Jingwen Zhang, Qing Yang, Kai Wei

**Affiliations:** 1 School of Marine Engineering, Dalian Maritime University, Dalian 116026, China; malaihao@dlmu.edu.cn; 2 School of Public Administration and Humanities, Dalian Maritime University, Dalian 116026, China; zhangjingwen2021@126.com (J.Z.); yangqing@dlmu.edu.cn (Q.Y.); weikai@dlmu.edu.cn (K.W.)

**Keywords:** lab safety, safety input, influencing factors, SEM, SD

## Abstract

Lab safety problems have become an impeding factor that cannot be ignored in normal teaching and scientific research activities at colleges and universities. As the risk factors of lab accidents can be conceptualized as occurring at multiple levels, systematically improving and optimizing lab safety is the crucial route to accident prevention in labs. In this paper, a novel method that integrates a structural equation model (SEM) and system dynamics (SD) is presented to dynamically assess lab safety with the characteristics of insufficient data and uncertainty. On the basis of a questionnaire investigation, the SEM was utilized to determine the influencing factors on lab safety and acquire the path coefficients among these factors, which were embedded into the SD model as the weight of the influencing factors. An illustration was carried out to test and validate the proposed method, and a sensitivity analysis was also conducted to recognize variables contributing the most to the improvement of lab safety. The results demonstrated that the safety input of human and management subsystems is the most effective to improve the lab safety; meanwhile, “safety awareness”, “emergency ability”, “operation skills”, “safety culture” and “safety training” are the top five contributing factors, which can promote lab safety in the shortest time.

## 1. Introduction

With the growth of academic institutions, the safety problems of laboratories at colleges and universities are becoming increasingly prominent [1]. A wide range of hazards, including chemical, inflammable and explosive materials, etc., are handled in the lab, making the lab personnel face significant threats at all levels in the dosage, usage and management of these hazard sources; even slight negligence in the process may cause accidents. Unfortunately, there is no authoritative and special database established by relevant parties to record lab accident and near-miss data [2]. However, some insights into lab accidents can be gained by using the partial statistical or regional data of lab accidents in open reports and the related literature, which is also enough to prove and demonstrate the severity of laboratory accident risk. According to statistics, there have been more than 260 accidents in chemical laboratories in the United States since 2001, most of which have caused casualties [3]. Recently, a few cases of lab accidents and the resulting injuries have aroused wide attention. An accident involving fire and an explosion happened in a laboratory at Tsinghua University on 18 December 2015, resulting in the death of a postdoctoral student on the spot [4]. Another case occurred in an environmental engineering laboratory at Beijing Jiaotong University on 26 December 2018; three students were killed in an explosion that occurred during a scientific research experiment on landfill leachate treatment [5]. According to the summary of 3 years of supervision of university laboratory safety organized by the Chinese Ministry of Education in 2015–2017, the 75 universities interviewed all had latent dangers in chemical safety management; 80% of the universities had hidden dangers in their laboratory water and electricity systems, and personal protection problems. The management of instruments and equipment in 76% of the universities was not standardized, and laboratory safety education and access in 45% of universities were defective [6]. In accordance with a report released by the Bureau of Labor Statistics, incidents are 11 times more likely to occur in chemical laboratories than in other types of laboratories [2]. Compared with industrial production safety, lab safety is not given top priority due to the perception that the small quantity of materials will not have a significant hazardous impact on people and the environment. However, the accidents highlighted above have provided a wake-up call to everybody and clearly highlight the need to improve accident prevention in laboratories.

Fortunately, lab safety has been focused on increasingly by laboratory researchers, governmental agencies, industries, universities and relevant parties in recent years. To explore the risk factors influencing laboratory safety and prevent laboratory accidents, many accidents’ causes and analysis models can be used to identify laboratory hazards. HAZOP (hazard and operability analysis) is the common and original method of lab safety management, which can help to allocate the measures according to the relative importance of risks [7,8,9,10,11]. Additionally, FMEA (failure mode effect and criticality analysis) [12,13], FTA (fault tree analysis) [14,15], ETA (event tree analysis) [16,17] and Bayesian networks (BNs) [3,13] have also been introduced into laboratory risk assessments. These various methods provide more options for risk identification from different perspectives [18,19,20]. However, hazards in the lab have different inherent characteristics compared with industry, e.g., high turnover of collaborators, students not being trained well for lab work, freedom of research, equipment often in the development stage and the difficulty of obtain accident statistics, etc. Hence, the risk analysis techniques commonly used as aforementioned in industry cannot directly migrate to the laboratories of colleges and universities [21,22]. Moreover, none of these approaches enable a dynamic and holistic analysis, thus failing to describe the variations in laboratory safety, which is a natural characteristic of laboratory safety management [23]. In addition, these models cannot provide predictions about safety states during the entire life cycle under different risk control measures, which is required for optimizing the risk control strategies [24]. As the risk factors of laboratory accidents can be conceptualized as occurring at multiple levels, how to systematically improve and optimize laboratory safety management effectiveness is the crucial route to accident prevention in laboratories [25]. Actually, a laboratory development can never be completely safe, but the degree of inherent safety can be increased by selecting the optimum design in terms of the human factors, equipment factors, environment factors and organizational management factors. However, although research on risk perception in laboratory safety has been paid more and more attention, as described above, to the best of our knowledge, there have been far fewer studies about implementing safety improvements in laboratories from the perspective of safety input–output. This is because the most challenging part of dynamic simulations for safety risks lies in the following: (1) identification of safety risk factors and their causal relationships, and (2) quantification of the variations in safety risk factors over the time.

To identify the influencing factors for laboratory safety and explore their contribution to laboratory safety improvement over time, in this paper, a methodology by integrating SEM and SD is proposed to measure the dynamic relationship between the influencing factors and laboratory safety levels from the perspective of safety input–output. 

## 2. Materials and Methods

### 2.1. Framework of Proposed Method

SD is a methodology and mathematical modeling technique that was put forward by Forrester for understanding and discussing complex issues and problems over time [26]. This method is a combination of theory and computer science, which has been used extensively to aid in maritime [27], project construction [28], education [29], resource and environment management [30], transportation planning [31] and many other fields. Owing to its outstanding advantages in simulating a dynamically complex system with internal feedback loops [32,33,34], it provides a path to solving the dynamic relationship between laboratory safety inputs and safety levels. Meanwhile, SD is based on a feedback loop, which is not sensitive to most non-key parameters in the model. As long as the estimation of the parameters is within a reasonable range, the model results will not show an unreasonable deviation. Therefore, the utilization of SD is helpful for overcoming the problems of risk uncertainty and insufficient data in laboratory safety assessments. Nevertheless, it is challenging to determine and quantify the variables and equations in the construction of an SD model. To address this challenge, a SEM model that is widely used for explaining the relationship between variables was introduced for the construction of an SD model. Compared with traditional multivariate regression models, SEM is more capable of testing the intrinsic structural relationships among variables in the model, and expressing these relationships in terms of causal models and path diagrams through factor analysis, path analysis and covariance analysis [35,36]. While SEM is a static research method, it is possible to build an SD model based on the correlations identified by the SEM. Therefore, an SD model based on the SEM was built in this paper to quantitatively predict the development trend of laboratory safety levels, as well as the degree of influence of the factors on laboratory safety.

**Figure 1:**
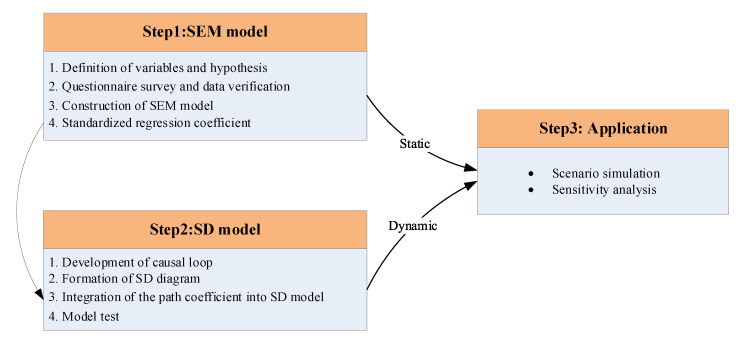
Framework and process of the proposed method.

As shown in Figure 1, this study was arranged into 3 steps. The first step involves the SEM modelling process, which includes definition of the latent variables and observed variables, the questionnaire survey and data verification, construction of the SEM model, and the output of path coefficients. Firstly, the key variables, including the latent variables and observed variables for indicating laboratory safety levels were identified based on expert interviews, accident analyses and a literature review. Secondly, a questionnaire based on the design variables was adopted to analyze the influencing factors for laboratory safety levels and their associated impact. Thirdly, after testing the validity and reliability of the questionnaire data, the SEM model was constructed by Amos software for factor analysis and correlation analysis. Finally, the path coefficients between the factors were produced as output after evaluating the goodness of fit of the initial SEM model.

The second step relates to the SD modeling process. In the process, based on the interactions among the variables, the causal loop that can help in understanding the interdependencies and feedback in laboratory safety was developed firstly. Secondly, the causal loop diagram was converted to a stock and flow diagram by using Vensim software, then the initial parameters and equations were assigned to the corresponding variables. Note that the normalized path coefficients of the latent variables and observed variables calculated in the SEM were converted to the weight of the factors in the SD model. Thirdly, the appropriateness of the linkages among variables was checked by a model validation test. 

The third step was the application of the SEM-SD model. Different scenarios were benchmarked to measure the influence of different safety inputs on laboratory safety levels in the dynamic simulation process.

### 2.2. Structural Equation Model (SEM)

#### 2.2.1. System Variables and Hypothesis

Regarding laboratory safety management as a system, the subsystems influencing the laboratory safety level can be summarized as the human subsystem, the equipment subsystem, the environment subsystem and the management subsystem, as determined by the related accident analysis and literature review. These subsystems were deemed to be latent variables in the structural model, and the corresponding observed variables were identified as influencing factors by 2 rounds of expert interviews, which involved laboratory teachers, managers and related safety experts. As a result, a total of 17 influencing factors as observed variables for the latent variables were obtained, and their symbols are listed in Table 1.

**Figure 2:**
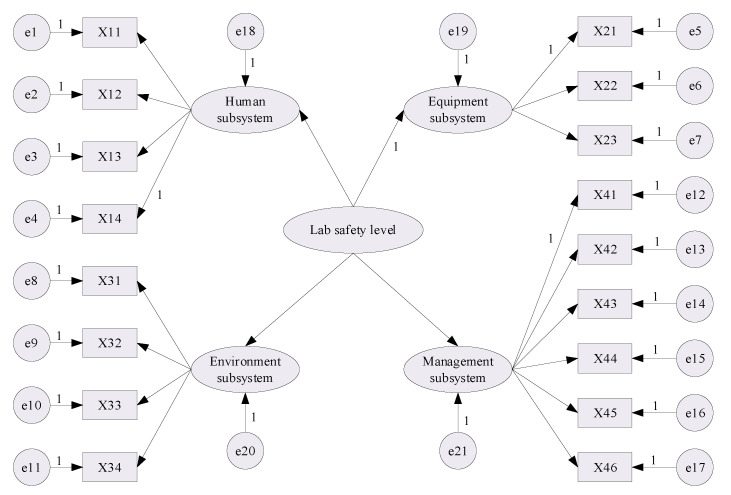
Initial SEM for laboratory safety level.

In this study, it was assumed that the human subsystem, the equipment subsystem, the environment subsystem and the management subsystem would have a significant impact on the laboratory safety level, but the correlation between these variables would be low. According to this hypothesis, the structural model concerning the influencing factors of the laboratory safety level is plotted as shown in Figure 2. In the Figure 2, e1~e17 represent the residual terms of the corresponding observed variable; e18–e21 express the residual terms of the corresponding latent variables. In the initial SEM, the regression weight of one variable in a group of variables was set to 1 first, so as to facilitate and support the SEM model’s operation [35].

#### 2.2.2. Questionnaire Survey and Data Verification

- Questionnaire survey

Based on the above definition of the latent variables and observed variables, a survey questionnaire involving each observation variable was developed to collect the data, which involved a variety of statements scored by the respondents on a 5-point Likert scale. As the questionnaire concerned the ranking of importance of the 17 observed variables in the SEM, the Likert scale method was used to measure the importance of different observed variables under the same latent variable, from low to high, earning scores of 1, 2, 3, 4, or 5. Respondents included students, teachers, technicians and administrators. In total, 180 questionnaires were distributed, of which 151 valid questionnaires were collected, giving rise to a response rate of 83.9%, which satisfied the investigation’s requirements. The characteristics of the effective sample are shown in Table 2, and the results of the questionnaire survey are summarized in Table 3.

- Reliability and validity verification

Reliability refers to the degree of consistency of the different respondents’ answers within the same questionnaire [37]. Cronbach’s alpha tests are widely used to assess the reliability of the scale items. The value of the Cronbach’s alpha coefficient is between 0 and 1. The higher the coefficient is, the more reliable the questionnaire is. Therefore, the Cronbach’s alpha coefficient was utilized to test the consistency of the latent variables in the questionnaire; the formula for its calculation is as follows [38]:

(1) $$\alpha =\frac{k}{k-1}\times \left(1-\frac{\sum_{i=1}^{k}Z_{i}^{2}}{Z_{T}^{2}}\right)$$

where k refers to the total number of items in the questionnaire, $Z_{i}^{2}$ denotes the variance within the score of the *i*^th^ question and $Z_{T}^{2}$ represents the variance of the total scores of all questions.

Validity is the correctness and quality of the questionnaire data, which refers to the degree to which the scale can be accurately measured. Validity analysis is generally expressed by the Kaiser–Meyer–Olkin (KMO) test and Bartlett’s test of sphericity [39]. KMO is used to test correlations between variables by comparing the correlation coefficients and partial correlation coefficients, and Bartlett’s test of sphericity is used to test whether the correlation coefficient matrix is the unit matrix. When the value of KMO is close to 1 and the significance probability of Bartlett’s test of sphericity is less than 0.05, this indicates that the survey data are suitable for factor analysis. The KMO can be calculated by the following formula [40]:

(2) $$KMO=\frac{\sum \sum_{i\neq j}r_{ij}^{2}}{\sum \sum_{i\neq j}r_{ij}^{2}+\sum \sum_{i\neq j}p_{ij}^{2}}$$

where $r_{ij}$ denotes the correlation coefficient between variable *i* and variable *j*, and $p_{ij}$ refers to the partial correlation coefficient between variable *i* and variable *j*.

In the present study, SPSS was used to test the reliability and validity of the measurement items in the SEM, with the analysis results being summarized in Table 4. It can be seen in the table that the reliability and validity of the present scale are acceptable for factor analysis.

#### 2.2.3. SEM Fit Evaluation 

After verification of the scale data, it was necessary to conduct a goodness of fit evaluation between the theoretical SEM and the data, aiming to ensure that the model had statistical operability and could output stable and reasonable results. Different fit indices can test the theoretical model from the perspective of model complexity, sample size, absoluteness and relativity, so a goodness of fit evaluation of a SEM cannot merely depend on a certain fitting index [41]. To this end, in this paper, the fit indices containing the absolute goodness of fit and the relative fitting index were utilized in combination to assess the initial model. The evaluation criteria of the goodness of fit are recapped in Table 5.

**Figure 3:**
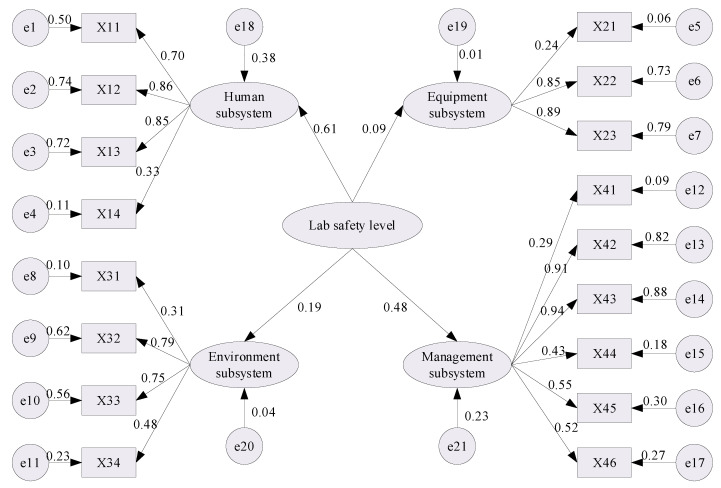
Standardized estimation results of Initial SEM.

After the raw survey data were imported into Amos software, the maximum likelihood estimation method was adopted to solve the SEM. The initial path correlation coefficients are depicted in Figure 3, and the corresponding goodness of fit indices used to test the fitting degree of the model structure are summarized in Table 6.

**Figure 4:**
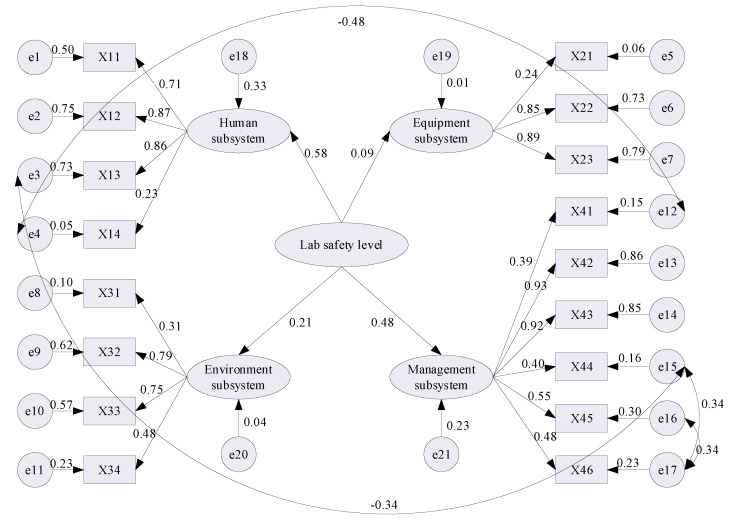
Standardized estimation results of the modified SEM.

As shown in Table 6, the RMSEA and NFI results of the initial SEM did not meet the model fitting requirements shown in Table 5. Therefore, it was necessary to modify the model according to the modification indices (M.I.) and parameter changes (Par Change), to improve the fitness of the model. In this paper, by adding the path relationships between the residual terms e3 and e15, between e4 and e12, between e15 and e17, and between e16 and e17, the modified standardized estimation results were eventually obtained, as shown in Figure 4; the modified SEM fit results are summarized as shown in Table 7, which indicates that the present model’s goodness of fit indices all satisfy the reference standard.

#### 2.2.4. Standardized Regression Coefficients

The normalized weights of the latent variables and observed variables were converted from the correlation path coefficients obtained from the modified SEM, which was used to establish following SD model, and the conversion results are listed in Table 8. Among the latent factors, the weight of the human subsystem is the largest, followed by the management subsystem, the environment subsystem and the equipment subsystem.

### 2.3. System Dynamics (SD)

#### 2.3.1. Development of the Causal Loop

**Figure 5:**
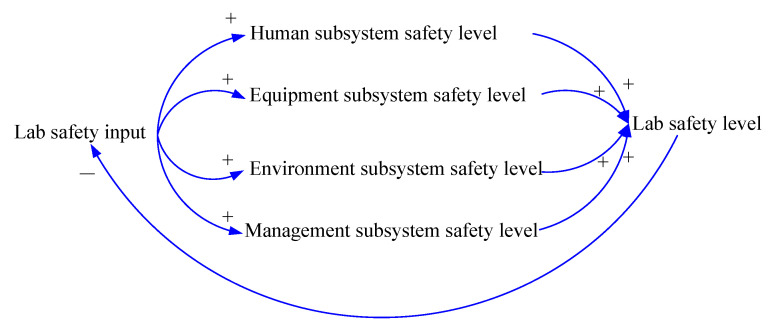
Causal loop of the laboratory safety management process.

A causal loop diagram is composed of one or more feedback loops, which reflect the relationship between the input and output of the factors in the system, on the one hand, and the relationship between the external environment and the input and output of the system on the other hand [42]. Safety input plays an important role in laboratory safety level. If the safety level is low, we should increase the laboratory safety input to improve the factors affecting safety. On the contrary, if the safety level is high, we can appropriately slow down the input into safety. In the present study, the internal causal relationships and evolution process among the influencing factors for laboratory safety were analyzed from the perspective of safety input–output. Based on the SEM, laboratory safety input was introduced to establish a causal loop that reflected the laboratory safety management process, as shown in Figure 5. It can be seen that laboratory safety input can promote the safety level of subsystems such as human factors, equipment factors, environmental factors and management factors, so as to improve the overall laboratory safety level. When the safety target level is reached, the laboratory safety input will be reduced through the feedback of the laboratory safety level to avoid wasting resources.

#### 2.3.2. Formation of the SD Diagram and Model Check 

**Figure 6:**
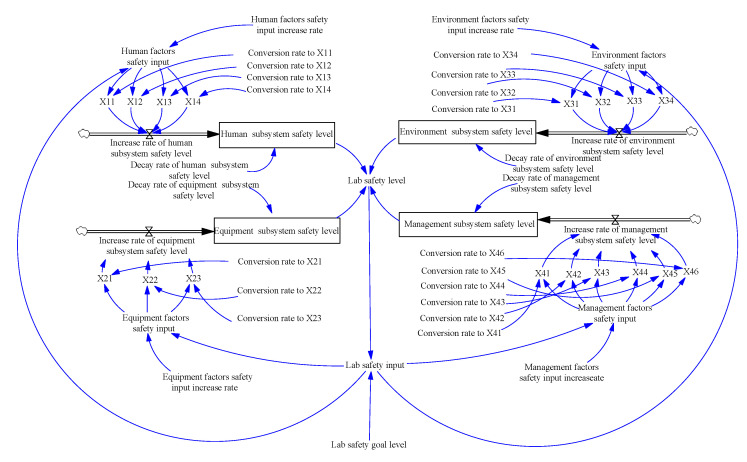
Stock and flow diagram of the SD model.

Once the causal loop was built, the stock and flow diagram could be created. Every causal loop in SD model should have at least one stock; otherwise, there will be no accumulation [43]. Only the flow can change the value of a stock, because all variables in the SD model change over time. On this basis, according to the causal loop diagram and the defined variables, the stock and flow diagram of the SD model for laboratory safety levels was obtained as shown in Figure 6, which includes 4 level variables, 4 rate variables, 21 auxiliary variables and 26 constants.

In Figure 6, the variables and corresponding equations that describe the system’s structure and govern their interrelationships among the various variables can be determined. Since the variables in the model are all qualitative, they were measured in dimensionless units. Noted that the SD equation of the latent and observed variables in SEM was edited with the weight of each factor as the coefficient. The mathematical expressions between the variables used in SD model are displayed in Table 9.

In the above equations, HSSL0, EqSSL0, EnSSL0, and MSSL0 indicate the initial values of the human subsystem’s safety level, the equipment subsystem’s safety level, the environment subsystem’s safety level and the management subsystem’s safety level, respectively. After determination of the equations, the next step was to check the SD model, including an operation check and a unit check, which were used to verify the rationality of causality, the accuracy of the equation and the consistency of the units. Through the function of the running check and the unit check in Vensim software, it was concluded that the SD model runs well. 

## 3. Results and Discussions

### 3.1. Model Test

**Figure 7:**
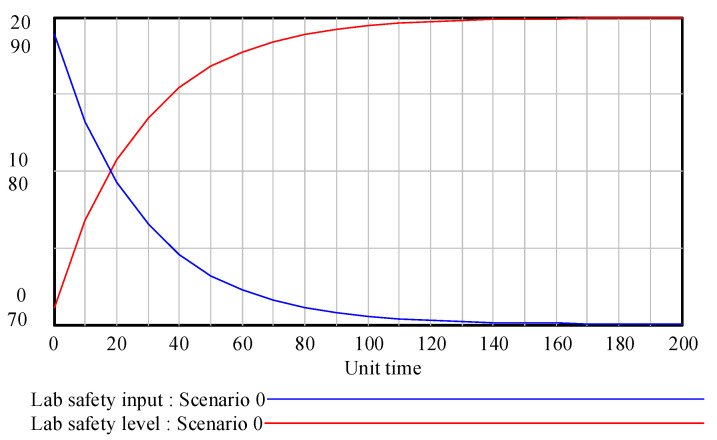
Dynamic relationship between laboratory safety level and safety input.

In this section, the developed methodology model was tested on Vensim software. The parameters of the established SD model can be divided into two categories: one is the initial value of the level variable; the other is the constant used for sensitivity adjustment. The initial value in the SD model can be determined by the expert scoring method. In terms of the level variables’ initial values, according to the 1–100 scoring system, the laboratory safety level was ranked as excellent (≥90), good (80–89), medium (70–79), passing (60–69) or poor (<60), which were used as the scoring standards. A survey was then carried out on 15 professionals, including professors, associate professors, managers of laboratories and safety research experts. As a result, the initial value of the human subsystem safety level, the equipment subsystem safety level, the environmental subsystem safety level and the management subsystem safety level were determined by the experts’ judgements, which were 70, 75, 75 and 70, respectively. In terms of the constants in the SD model, to perform the sensitivity analysis, the decay rates were set at 0.001, the safety input increase rates were set at 0.3 and the conversion rates were set at 0.1. The above equations and parameters were substituted into the established SD model, the simulation time was set to 200 units of time, and the laboratory safety goal level was set to 90. After that, the simulation results were obtained by running Vensim software. The dynamic relationship between laboratory safety level and safety input is displayed in Figure 7.

In Figure 7, under the given conditions of laboratory safety input, the initial value of laboratory safety level is about 71.1. With the effect of safety input on the human factors, equipment factors, environmental factors and management factors, the overall laboratory safety level increased rapidly and then slowed down, coming close to the target safety level of 90 by the 140th unit of time. If the laboratory safety level does not reach the safety goal level, the SD model will adjust the safety input according to the deviation between the laboratory safety target and the actual safety level. With improvement in the laboratory safety level, the corresponding safety input will gradually reduce. When the laboratory safety level reaches the target level, its growth is not zero, which is due to the time delay between the laboratory safety input and the laboratory safety level.

### 3.2. Scenario Simulation and Sensitivity Analysis

**Figure 8:**
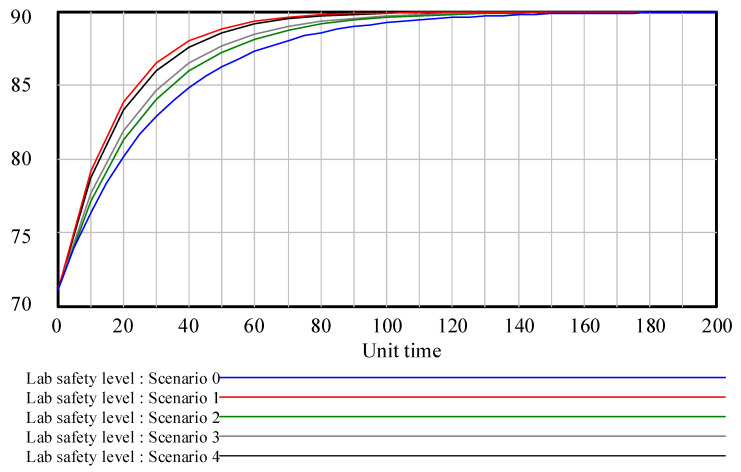
Laboratory safety level with different safety input increase rates.

In this section, the established SD model was utilized to simulate the contribution of the laboratory safety subsystems and the corresponding influencing factors to the laboratory safety level. Firstly, to observe the contribution of different safety inputs of the laboratory safety subsystems to laboratory safety level, the increase rates of safety input were set as shown in Table 10; the corresponding results of laboratory safety level under different input increase rates for each subsystem are depicted in Figure 8.

As can be seen from Figure 8, increasing the safety input of any subsystem will improve the laboratory safety level, but the response rate of laboratory safety level is different. Among the subsystems, the safety input into the human factors presented the largest growth rate for improving the laboratory safety level, followed by management factors. To perform the sensitivity analysis and recognize the most highly contributing factors in laboratory safety, the contribution rate (CR) was defined in this paper; the CR for the influencing factors for laboratory safety management were calculated with Equation (3).

(3) $$CR\left(A_{i}\right)=\frac{\bar{LSL\left(X_{i}\right)}-\bar{LSL\left(X_{0}\right)}}{\bar{LSL\left(X_{0}\right)}}$$

where $\bar{LSL\left(X_{0}\right)}$ denotes the average laboratory safety level affected by the influencing factor *X* before changing it and $\bar{LSL\left(X_{i}\right)}$ refers to the average laboratory safety level after changing the influencing factor *X*.

**Figure 9:**
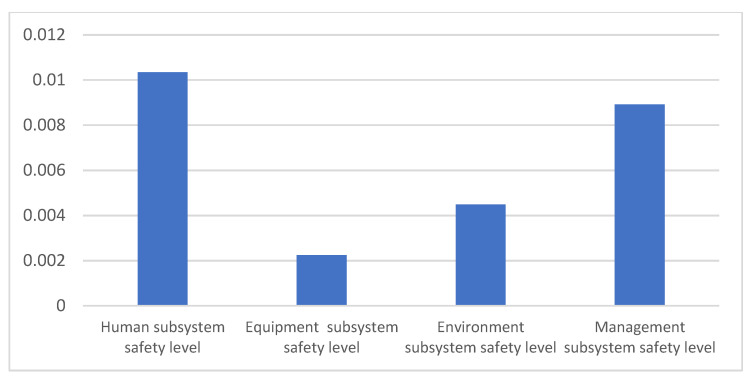
CR results of the subsystems on laboratory safety levels.

As provided in Equation (3), CR refers to the average increased percentage of the laboratory safety level when one factor increased by a certain value while the other factors remain unchanged. Compared with the weight of factors, the CR can quantitatively express the contribution that the changes in a certain factor make to the laboratory safety level, which can better provide evidence for decision-making regarding laboratory safety inputs. Based on the simulation results, the CR for each subsystem in laboratory safety management could be worked out with Equation (3), as shown in Figure 9. The CRs of “Human subsystem safety level” and “Management subsystem safety level” were apparently higher than those of the other two subsystems regarding laboratory safety under the same safety input increase rate, indicating that these two subsystems are the most critical aspects contributing to improvement of the laboratory safety level.

**Figure 10:**
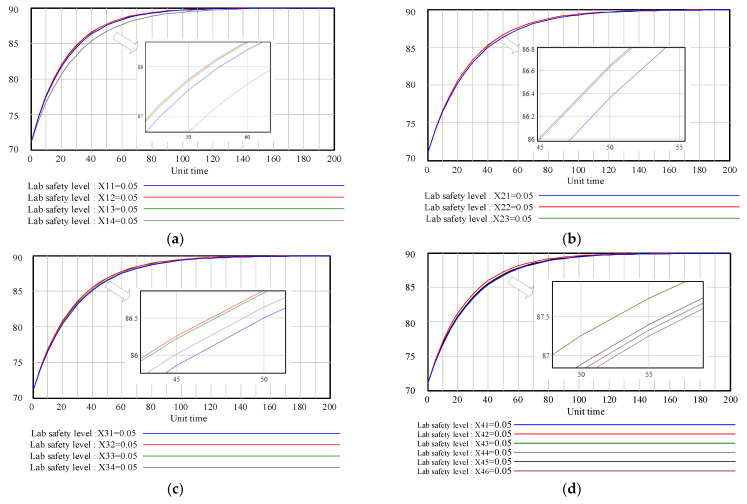
Laboratory safety level with different conversion rates: (**a**) human factors; (**b**) equipment factors; (**c**) environment factors; (**d**) management factors.

Different conversion rates of the influencing factors are related to the laboratory risk control measures. To further analyze the contribution of different control measures on the laboratory safety level, likewise, each conversion rate increased by 50% in turn, while the other variables remained unchanged (the initial value was 0.1). The response of the corresponding laboratory safety level is shown in Figure 10.

According to Figure 10, the same increase in the conversion rates of the influencing factors could improve the laboratory safety level at different rates. For example, in terms of the human factors subsystem, an improvement in the conversion rate of “Safety awareness” and “Emergency ability” could promote the laboratory safety in the shortest time.

**Figure 11:**
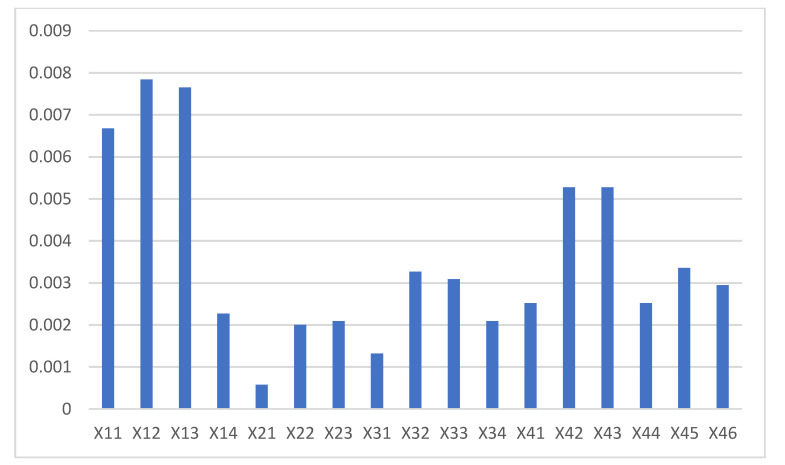
CR results of the influencing factors regarding laboratory safety levels.

The sensitivity of each factor to laboratory safety level was also determined according to Equation (3). The CR results of each influencing factor regarding the laboratory safety level are provided in Figure 11. It can be concluded that “Safety awareness”, “Emergency ability”, “Operation skills”, “Safety culture” and “Safety training” are the top five contributing factors to the laboratory safety level, namely, an increase in the conversion rate of these influencing factors would make the greatest contribution to the improvement of laboratory safety levels. Therefore, although the safety input of the human and management subsystems is the most effective means to improve the laboratory safety level, as the results in Figure 9 show, the conversion rate of these factors should deserve enough attention at the same time when making laboratory safety input decisions. This is because improving the conversion rate of these factors in the human and management subsystems can promote better laboratory safety levels in the shortest time.

### 3.3. Comparison with Other Similar Studies

The value of this paper is in the development of an approach combining SEM and SD models to address the dynamic relationships of laboratory safety management elements within a single framework. Although there are a few studies on laboratory safety improvements from the perspective of safety input–output, several studies on laboratory safety risk assessment can be used for comparative analysis. From a statistical perspective, improper storage and handling comprised the most frequent human cause for laboratory incidents (27%), followed by procedure violations (7%) [2]; these two human factors can be considered as aspects of “Operation skills” in the present study. Lack of professional knowledge and not receiving professional training were recognized as the most important events in the laboratory explosion accidents by using FTA [17], which consistent with the results in the present study. In a smaller survey on 85 respondents, the results showed that 9% did not know how to handle an emergency in the laboratory fire and explosion accidents [44], which indicates that emergency ability is also a crucial factor in improving laboratory safety. Furthermore, the most important reason for the existence of these pervasive risk factors in the laboratory is the lack of strong safety awareness and a positive safety culture [1]. Different from industry, the barrier to establishing and boosting a laboratory safety culture is academic freedom, which is often raised as an objection to safety practices in the laboratory.

## 4. Conclusions

An integrated model based on SEM and SD was proposed in this paper to identify the factors affecting laboratory safety effectiveness, and to measure the contribution to laboratory safety improvements over time from the perspective of safety input–output. The main conclusions are summarized as follows:

(1) The proposed model was proven to be an effective method for laboratory safety assessments. With this model, the dynamical evolution of the laboratory safety level over time can be simulated under different scenarios.
(2) The influencing factors of laboratory safety were analyzed from four aspects, including the human subsystem, the equipment subsystem, the environment subsystem and the management subsystem. The responses to safety inputs into the four subsystems of the laboratory safety level are different: the human and management subsystems are the most effective for improve the laboratory safety level.
(3) “Safety awareness”, “Emergency ability”, “Operation skills”, “Safety culture” and “Safety training” were demonstrated to be the top five factors contributing to the laboratory safety level, which can promote a better laboratory safety level in the shortest time.

The simulation results in the present study have reference value for laboratory safety risk assessment and management. Meanwhile, since the best laboratory safety input scheme can be determined by adjusting the safety devotion and conversion rate of the influencing factors, the proposed model may have guiding significance for making laboratory safety input and management decisions. As the human and management factors are the most effective for improve the laboratory safety level, in the future, potential work should be devoted to investigating the causal relationships between laboratory safety and human and organizational factors, and to establishing effective measures for alleviating human and organizational failures in the laboratory at colleges and universities.

## Figures and Tables

**Table 1:** Latent and observed variables in the SEM.

Latent Variable	Observed Variable	Symbols
Human subsystem	Operation skills	X11
	Safety awareness	X12
	Emergency ability	X13
	Psychological quality	X14
Equipment subsystem	Safety protection devices	X21
	Personal protective equipment (PPE)	X22
	Fire control facilities	X23
Environment subsystem	Space layout	X31
	Sanitary conditions	X32
	Warning signs	X33
	Ventilation	X34
Management subsystem	Equipment maintenance	X41
	Safety culture	X42
	Safety training	X43
	Management of hazardous chemicals	X44
	Safety checks	X45
	Access management	X46

**Table 2:** Basic information of questionnaire respondents.

Name	Category	Number of People	Percentage
Gender	Man	101	66.89%
	Woman	50	33.11%
Position	Student	60	39.74%
	Teacher	31	20.53%
	Technician	35	23.18%
	Manager	24	15.89%
Age	20–30	65	43.05%
	31–40	42	27.81%
	41–50	31	20.53%
	51–60	13	8.61%

**Table 3:** Summary of the results of the questionnaire survey.

Score						Score					
Item	1	2	3	4	5	Item	1	2	3	4	5
X11	2	10	11	73	55	X33	4	9	22	67	49
X12	2	10	30	53	56	X34	4	25	48	44	30
X23	1	11	23	49	67	X41	4	53	65	9	20
X14	1	8	18	57	67	X42	1	8	23	67	52
X21	0	5	35	62	49	X43	1	8	24	70	48
X22	2	27	52	34	36	X44	5	12	38	67	29
X23	2	38	49	35	27	X45	1	8	36	60	46
X31	0	19	32	57	43	X46	4	7	32	59	49
X32	4	3	22	75	47						

**Table 4:** The reliability and validity results.

	Cronbach’s α	KMO of Sampling Adequacy	Bartlett’s Test of Sphericity	
			Approx. Chi-Square	Sig.
Standard	>0.9 as excellent	>0.9 as excellent	NA	<0.05
	0.7–0.8 as acceptable range	0.6~0.8 as acceptable range		
Results	0.726	0.652	1039.175	0.000

**Table 5:** Fit index evaluation standards [35].

**Index Name**	**Statistical Test**	**Standard or Critical Value of Fit**
Absolute goodness of fit	*χ*^2^/df (CMIN/DF)	3 ≤ *χ*^2^/df < 5 as acceptable range
		*χ*^2^/df ≤ 3 = model fitting degree is excellent
	RMSEA (estimated root mean square)	0.05 ≤ RMSEA ≤ 0.1 as acceptable range
		RMSEA < 0.05 height fitting model
Relative fitting index	NFI (normal fit index)	NFI > 0.8 as acceptable range
		NF1 > 0.9 = model fitting degree is good
	IFI (incremental fit index)	IFI > 0.8 as acceptable range
		IFI > 0.9 = model fitting degree is good
	CFI (comparative fit index)	CFI > 0.8 as acceptable range
		CFI > 0.9 = model fitting degree is good

**Table 6:** Initial SEM fit results.

**Index**	**χ2/df**	**RMSEA**	**NFI**	**IFI**	**CFI**
Results	2.597	0.103	0.725	0.811	0.807
	excellent	unacceptable	unacceptable	acceptable	acceptable

**Table 7:** Modified SEM fit results.

**Index**	**χ2/df**	**RMSEA**	**NFI**	**IFI**	**CFI**
Results	1.916	0.078	0.804	0.896	0.893
	excellent	acceptable	acceptable	acceptable	acceptable

**Table 8:** Standardized regression coefficients and corresponding normalized weights.

**Path Relation**	**Standard Regression Coefficient**	**Weight**
Laboratory safety level → Human subsystem	0.58	0.43
Laboratory safety level → Equipment subsystem	0.09	0.07
Laboratory safety level → Environment subsystem	0.21	0.15
Laboratory safety level → Management subsystem	0.48	0.35
Human subsystem → X11	0.71	0.27
Human subsystem → X12	0.87	0.33
Human subsystem → X13	0.86	0.32
Human subsystem → X14	0.23	0.08
Equipment subsystem → X21	0.24	0.12
Equipment subsystem → X22	0.85	0.43
Equipment subsystem → X23	0.89	0.45
Environment subsystem → X31	0.31	0.13
Environment subsystem → X32	0.79	0.34
Environment subsystem → X33	0.75	0.32
Environment subsystem → X34	0.48	0.21
Management subsystem → X41	0.39	0.11
Management subsystem → X42	0.93	0.25
Management subsystem → X43	0.92	0.25
Management subsystem → X44	0.40	0.11
Management subsystem → X45	0.55	0.15
Management subsystem → X46	0.48	0.13

**Table 9:** Variables and functions in the SD model.

Variable	Type	Symbol	Function
Laboratory safety level	Auxiliary	LSL	LSL = 0.43×HSSL + 0.07 × EqSSL + 0.15 × EnSSL + 0.35×MSSL
Laboratory safety goal level	Constant	LSGL	NA
Laboratory safety input	Auxiliary	LSI	LSI = LSGL − LSL
Human subsystem safety level	Level	HSSL	HSSL = INTEG (IRH − DRH, HSSL0)
Decay rate of the human subsystem safety level	Constant	DRH	NA
Increase rate of the human subsystem safety level	Rate	IRH	IRH = 0.27 × X11 + 0.33 × X12 + 0.32 × X13 + 0.08 × X14
Human factors’ safety input	Auxiliary	HFSI	HFSI = LSI × HFIR
Human factors’ safety input increase rate	Constant	HFIR	NA
Conversion rate to X1*i*, *i* = 1, 2, 3, 4	Constant	CRX1*i*	NA
X1*i*, *i* = 1, 2, 3, 4	Auxiliary	X1*i*	X1*i =* CRX1*i* × HFSI
Equipment subsystem safety level	Level	EqSSL	EqSSL = INTEG (IREq − DREq, EqSSL0)
Decay rate of the equipment subsystem safety level	Constant	DREq	NA
Increase rate of the equipment subsystem safety level	Rate	IREq	IREq = 0.12 × X21 + 0.43 × X22 + 0.45 × X23
Equipment factors’ safety input	Auxiliary	EqFSI	EqFSI = LSI × EqFIR
Equipment factors’ safety input increase rate	Constant	EqFIR	NA
Conversion rate to X2*i*, *i* = 1, 2, 3	Constant	CRX2*i*	NA
X2*i*, *i* = 1, 2, 3	Auxiliary	X2*i*	X2*i =* CRX2*i* × EqFSI
Environmental subsystem safety level	Level	EnSSL	EnSSL = INTEG (IREn − DREn, EnSSL0)
Decay rate of the environmental subsystem safety level	Constant	DREn	NA
Increase rate of the environmental subsystem safety level	Rate	IREn	IREn = 0.13 × X31 + 0.34 × X32 + 0.32 × X33 + 0.21 × X34
Environmental factors’ safety input	Auxiliary	EnFSI	EnFSI = LSI × EnFIR
Environmental factors’ safety input increase rate	Constant	EnFIR	NA
Conversion rate to X3*i*, *i* = 1, 2, 3, 4	Constant	CRX3*i*	NA
X3*i*, *i* = 1, 2, 3, 4	Auxiliary	X3*i*	X3*i =* CRX3*i* × EnFSI
Management subsystem safety level	Level	MSSL	MSSL = INTEG (IRM – DRM, MSSL0)
Decay rate of the management subsystem safety level	Constant	DRM	NA
Increase rate of the management subsystem safety level	Rate	IRM	IRM = 0.11 × X41 + 0.25 × X42 + 0.25 × X43 + 0.11 × X44 + 0.15 × X45 + 0.13 × X46
Management factors’ safety input	Auxiliary	MFSI	MFSI = LSI × MFIR
Management factors’ safety input increase rate	Constant	MFIR	NA
Conversion rate to X4*i*, *i* = 1, 2, 3, 4, 5, 6	Constant	CRX4*i*	NA
X4*i*, *i* = 1, 2, 3, 4, 5, 6	Auxiliary	X4*i*	X4*i =* CRX4*i* × MFSI

**Table 10:** Different safety input scenarios.

Scenario	HFIR	EqFIR	EnFIR	MFIR
Scenario 0	0.3	0.3	0.3	0.3
Scenario 1	0.6	0.3	0.3	0.3
Scenario 2	0.3	0.6	0.3	0.3
Scenario 3	0.3	0.3	0.6	0.3
Scenario 4	0.3	0.3	0.3	0.6

## Data Availability

The data that support the findings of this study are available from the corresponding author upon reasonable request.
